# Randomised trial of genetic testing and targeted intervention to prevent the development and progression of Paget’s disease of bone

**DOI:** 10.1136/ard-2023-224990

**Published:** 2023-12-20

**Authors:** Jonathan Phillips, Deepak Subedi, Steff C Lewis, Catriona Keerie, Owen Cronin, Mary Porteous, David Moore, Roseanne Cetnarskyj, Lakshminarayan Ranganath, Peter L Selby, Tolga Turgut, Geeta Hampson, Rama Chandra, Shu Ho, Jon Tobias, Steven Young-Min, Malachi J McKenna, Rachel K Crowley, William D Fraser, Jonathan C Y Tang, Luigi Gennari, Rannuccio Nuti, Maria Luisa Brandi, Javier Del Pino-Montes, Jean-Pierre Devogelaer, Anne Durnez, Giovanni Carlo Isaia, Marco Di Stefano, Nuria Guanabens, Josep Blanch Rubio, Markus J Seibel, John P Walsh, Sarah L Rea, Mark A Kotowicz, Geoffrey C Nicholson, Emma L Duncan, Gabor Major, Anne Horne, Nigel Gilchrist, Stuart H Ralston

**Affiliations:** 1 Centre for Genomic and Experimental Medicine, Institute of Genetics and Cancer, Western General Hospital, University of Edinburgh, Edinburgh, UK; 2 Department of Radiology and Nuclear Medicine, Western General Hospital, Edinburgh, UK; 3 Edinburgh Clinical Trials Unit, The Usher Institute, The University of Edinburgh, Edinburgh, UK; 4 Rheumatic Diseases Unit, Western General Hospital, Edinburgh, UK; 5 School of Medicine, University College Cork, University College Cork, National University of Ireland, Cork, Ireland; 6 South East Scotland Molecular Genetics Service, NHS Lothian, Edinburgh, UK; 7 School of Health Sciences, University of Dundee, Dundee, UK; 8 Clinical Biochemistry and Metabolic Medicine, University of Liverpool, Liverpool, UK; 9 Department of Diabetes, Endocrinology and Metabolism, Manchester Royal Infirmary, Manchester, UK; 10 Clinical Genetics, Manchester Centre for Genomic Medicine, Manchester University Hospitals Foundation NHS Trust, Manchester, UK; 11 Department of Chemical Pathology, St Thomas' Hospital, London, UK; 12 King's College Hospital, London, UK; 13 Rheumatology, Robert Jones and Agnes Hunt Orthopaedic and District Hospital NHS Trust, Oswestry, UK; 14 Shrewsbury and Telford Hospital NHS Trust, Shrewsbury, UK; 15 Musculoskeletal Research Unit, Translational Health Sciences, Bristol Medical School, University of Bristol, Bristol, UK; 16 Queen Alexandra Hospital, Portsmouth, UK; 17 Department of Endocrinology and Diabetes Mellitus, St Vincent's University Hospital, Dublin, Ireland; 18 Rare Disease Clinical Trial Network, University College Dublin, Dublin, Ireland; 19 Norwich Medical School, University of East Anglia, Norwich, UK; 20 Departments of Endocrinology and Clinical Biochemistry, University of East Anglia, Norwich, UK; 21 Department of Medicine, Surgery and Neurosciences, University of Siena, Siena, Italy; 22 FIRMO Foundation, Florence, Italy; 23 Bone Centre, Università Vita-Salute San Raffaele, Milan, Italy; 24 Rheumatology, University of Salamanca, Salamanca, Spain; 25 Department of Rheumatology, Saint-Luc University Hospital, Université catholique de Louvain, Brussels, Belgium; 26 Department of Rheumatology, AZ Jan Portaels Hospital, Vilvoorde, Belgium; 27 University of Turin, Turin, Italy; 28 Department of Rheumatology, Hospital Clinic, IDIBAPS, University of Barcelona, Barcelona, Spain; 29 Rheumatology Department, Hospital del Mar, Barcelona, Spain; 30 Concord Repatriation General Hospital, Concord, New South Wales, Australia; 31 Department of Endocrinology and Diabetes, Sir Charles Gairdner Hospital, Nedlands, Western Australia, Australia; 32 Centre for Molecular Medicine and Innovative Therapeutics, Health Futures Institute, Murdoch University, Perth, Western Australia, Australia; 33 Perron Institute for Neurological and Translational Science, Perth, Western Australia, Australia; 34 Institute for Mental and Physical Health and Clinical Translation, Deakin University, Geelong, Victoria, Australia; 35 Department of Medicine at Western Health, The University of Melbourne, St Albans, Victoria, Australia; 36 University Hospital Geelong, Barwon Health, Geelong, Victoria, Australia; 37 Rural Clinical School, The University of Queensland, Toowoomba, Queensland, Australia; 38 Endocrinology Department, Royal Brisbane and Women's Hospital, Herston, Queensland, Australia; 39 School of Life Course & Population Sciences, Faculty of Life Sciences and Medicine, King’s College London, London, UK; 40 Department of Endocrinology, Guy’s and St Thomas’ NHS Foundation Trust, London, UK; 41 Rheumatology, John Hunter Hospital, New Lambton Heights, New South Wales, Australia; 42 Faculty of Medicine, The University of Newcastle, Callaghan, New South Wales, Australia; 43 Department of Medicine, The University of Auckland, Auckland, New Zealand; 44 Princess Margaret Hospital, Christchurch, New Zealand

**Keywords:** Patient Reported Outcome Measures, Pharmacogenetics, Therapeutics

## Abstract

**Introduction:**

Paget’s disease of bone (PDB) frequently presents at an advanced stage with irreversible skeletal damage. Clinical outcomes might be improved by earlier diagnosis and prophylactic treatment.

**Methods:**

We randomised 222 individuals at increased risk of PDB because of pathogenic *SQSTM1* variants to receive 5 mg zoledronic acid (ZA) or placebo. The primary outcome was new bone lesions assessed by radionuclide bone scan. Secondary outcomes included change in existing lesions, biochemical markers of bone turnover and skeletal events related to PDB.

**Results:**

The median duration of follow-up was 84 months (range 0–127) and 180 participants (81%) completed the study. At baseline, 9 (8.1%) of the ZA group had PDB lesions vs 12 (10.8%) of the placebo group. Two of the placebo group developed new lesions versus none in the ZA group (OR 0.41, 95% CI 0.00 to 3.43, p=0.25). Eight of the placebo group had a poor outcome (lesions which were new, unchanged or progressing) compared with none of the ZA group (OR 0.08, 95% CI 0.00 to 0.42, p=0.003). At the study end, 1 participant in the ZA group had lesions compared with 11 in the placebo group. Biochemical markers of bone turnover were significantly reduced in the ZA group. One participant allocated to placebo required rescue therapy with ZA because of symptomatic disease. The number and severity of adverse events did not differ between groups.

**Conclusions:**

Genetic testing for pathogenic *SQSTM1* variants coupled with intervention with ZA is well tolerated and has favourable effects on the progression of early PDB.

**Trial registration number:**

ISRCTN11616770.

**WHAT IS ALREADY KNOWN ON THIS TOPIC**

- Paget’s disease of bone (PDB) frequently presents at an advanced stage with complications secondary to irreversible skeletal damage. Clinical outcomes might be improved by earlier diagnosis and prophylactic treatment.
- Genetic factors are important in the pathogenesis of PDB and individuals who carry pathogenic variants in *SQSTM1* have more severe disease with an earlier age at onset than those who do not.

**WHAT THIS STUDY ADDS**

- Genetic testing for pathogenic *SQSTM1* variants in people with a family history of PDB coupled with radionuclide bone scan examination in those that test positive can be used to detect the disease at an early stage.
- Prophylactic treatment with zoledronic acid (ZA) in *SQSTM1* positive individuals favourably affects the development and progression of early Paget’s disease.

**HOW THIS STUDY MIGHT AFFECT RESEARCH, PRACTICE OR POLICY**

- This study supports the introduction of a programme of genetic testing for people with a family history of PDB coupled with the offer of prophylactic ZA treatment in carriers of pathogenic *SQSTM1* variants.

## Introduction

Paget’s disease of bone (PDB) is characterised by focal increases in bone remodelling at one or more skeletal sites.^1^ These abnormalities can cause various complications including bone pain, deformity, pathological fractures, nerve compression syndromes, deafness, secondary osteoarthritis and osteosarcoma.^2^ Bisphosphonates are highly effective at reducing raised bone turnover in PDB and are the treatment of choice for pain control,^3^ but clinical and symptomatic responses to treatment are blunted in patients with advanced disease who have already developed skeletal damage.^4 5^ Genetic factors play a key role in PDB, and the most important susceptibility gene is *SQSTM1*.^6 7^ Pathogenic variants in this gene have been detected in between 40% and 50% of people with a family history of PDB and up to 15% of those who are unaware of a family history of the disease.^8^ Cross-sectional studies have reported that carriers of pathogenic *SQSTM1* variants have more severe disease with an earlier age at onset than those that do not carry such variants^9^; and that up to 80% of pathogenic *SQSTM1* variant carriers develop PDB by the seventh decade.^8^ Very little is known about the natural history of PDB in carriers of pathogenic *SQSTM1* variants and is also unclear whether early therapeutic intervention might exert favourable effects on the evolution of PDB in these individuals. Here, we evaluated the acceptability of a programme of genetic testing for pathogenic *SQSTM1* variants in people with a family history of PDB, coupled with an invitation to enrol into a double blind, placebo-controlled randomised trial in which carriers of pathogenic *SQSTM1* variants were treated with a single infusion of 5 mg zoledronic acid (ZA) or placebo.

## Methods

### Trial design and oversight

The ZA to prevent the development of Paget’s disease (ZiPP) study was an investigator-led, multicentre, randomised, placebo-controlled trial conducted in 25 centres from 7 countries worldwide. The protocol for the study has previously been published.^10^ Participants were randomised in a 1:1 ratio to receive ZA or placebo. Enrolment commenced in March 2010, closed in April 2015 and follow-up was completed by December 2021 (online supplemental table 1).

10.1136/ard-2023-224990.supp1 Supplementary data

### Participants

Individuals who tested positive for pathogenic *SQSTM1* variants through a programme of genetic testing described previously^10^ were eligible to participate if they were 30 years of age or older; had not been diagnosed with PDB and had no contraindication to receiving ZA. Individuals who had currently or previously been treated with bisphosphonates were excluded from participating in the study.

### Setting

The trial was primarily based in secondary care referral centres where participants were recruited through family members (probands) who already had been diagnosed with PDB. The study involved an initial phase of genetic screening to identify eligible participants. Patients with PDB attending outpatient clinics (n=1428) underwent genetic testing for pathogenic *SQSTM1* variants according to standard techniques. If the result was positive, 1307 first-degree relatives of these individuals (primarily children) were offered genetic testing. Individuals who consented to undergo testing (n=750) and were found to be positive for pathogenic *SQSTM1* variants (n=350) were eligible to take part and 222 consented to enrol in the study. Details of the recruitment sites and names of the principal investigators at each site are shown in online supplemental table 2.

### Sample size

The sample size was chosen assuming that 15% of patients in the placebo group and 1.5% of patients in the active (ZA) treatment group would develop new PDB-like bone lesions during follow-up. This estimate of progression of lesions in the placebo group was based on previous cross-sectional studies.^11^ The effect size of the intervention was based on the observation that ZA has been reported to normalise biochemical markers of bone turnover for up to 6.5 years in 90% of patients with established PDB^12^ With this assumption, 85 subjects in each group would provide 89% power to detect a treatment effect of this magnitude at an alpha of 0.05. Since it is possible that more than one affected subject per family could be enrolled, the sample size was inflated to account for relatedness of individuals. This was done by calculating the mean squared alkaline phosphatase values in patients within families who carried the same mutation (271.3) and the mean squared alkaline phosphatase values between families (619.7) and combining this with an estimate that two subjects per family may be enrolled in the study, resulting in a design effect factor of 1.39, inflating the required sample size to 118 per group. The sample size was further inflated to account for a 10% rate of participants lost to follow-up resulting in a total sample size of 130 subjects per group or 260 subjects in total. The actual number of subjects randomised to the interventional study by the time recruitment had closed in April 2015 was 222. The decision to stop recruitment was based on funding and justified by recalculating the design factor based on the actual number of subjects per family that had been enrolled into the study (1.5 on average) yielding a revised design factor of 1.26.

### Randomisation and interventions

Randomisation used a web-based system hosted by Edinburgh Clinical Trials Unit, which used minimisation for variables thought to influence the occurrence of PDB including: the type of variant (missense vs truncating or frameshift); gender; whether the baseline radionuclide bone scan had shown lesions thought to be suggestive of PDB; whether serum alkaline phosphatase levels at baseline were elevated (yes/no); and by age band. Following randomisation, the study database generated a treatment code which was used by the research pharmacies at participating centres to dispense the investigational medical product. The intervention was a single intravenous infusion of 5 mg ZA administered over a 15 min period or a placebo that looked identical.

### Outcomes

The primary outcome was the proportion of participants who developed new bone lesions with the characteristics of PDB as assessed by radionuclide bone scan. The secondary outcomes comprised the number of new bone lesions and change in activity of existing bone lesions as assessed by semiquantitative analysis of bone scans by observers blinded to treatment allocation; the number of skeletal events related to PDB; biochemical markers of bone remodelling; health related quality of life assessed by the short form 36^13^ (SF-36); the presence and location of musculoskeletal pain assessed by the brief pain inventory^14^ (BPI); and anxiety and depression assessed by the Hospital Anxiety and Depression Score (HADS). Data on adverse effects and serious adverse effects were collected throughout the study. Full details of the genetic testing phase and the protocol have previously been published,^10^ but the protocol and statistical analysis plan are available as Online supplemental material on the journal website.

### Skeletal imaging and analytical methods for biochemical markers of bone turnover

Radionuclide bone scintigraphy with 99^m^Tc labelled bisphosphonate was performed at the baseline visit and at the end of study according to standard techniques at the participating centres. Anonymised images were assessed independently by imaging experts (DS and SHR) blinded to treatment allocation for evidence of bone lesions with the characteristics of PDB. At the end of study, a semiquantitative assessment was performed where a record was made of whether existing lesions present at baseline had disappeared, were thought to have reduced in intensity, remained the same, had worsened or whether new lesions had developed. New lesions, worsening lesions and no change in bone scan appearances were considered to indicate a poor outcome. The rationale for considering no change as a poor outcome was because continued evidence of tracer uptake in an affected bone indicates that the disease had remained metabolically active.

### Biochemistry

Biochemical measurements were made at baseline, at annual visits and at the end of study on blood samples collected between 09:00 and 12:00 hours after an overnight fast. Routine biochemical analysis of safety bloods and total alkaline phosphatase were performed by the local hospital laboratories. Specialised biochemical markers of bone turnover were measured centrally at the University of East Anglia at baseline, annual review visits and at the end of study visit. They comprised type I collagen C-terminal telopeptides (CTX) as a marker of bone resorption; procollagen type I amino-terminal propeptides (PINP) and serum bone-specific alkaline phosphatase (BAP) as markers of bone formation. Full details of the assays employed their precision and the reference ranges in men and women are provided in online supplemental material available on the journal website.

### Genetic analysis

Genetic testing for *SQSTM1* variants was carried out by Sanger sequencing of exons 7 and 8 of *SQSTM1* and the intron–exon boundaries gene according to standard techniques. Pathogenicity was assessed by the UK Association for Clinical Genomic Science (ACGS) best practice guidelines for variant classification in rare disease,^15^ which in turn were based on the consensus recommendations of the American College of Medical Genetics and Genomics and the Association for Molecular Pathology^16^

### Statistical analysis

The principal analysis was based on an intention-to-treat principle incorporating all randomised participants, regardless of treatment received. Due to the small number of events for the primary outcome, a Fisher’s exact test was performed to evaluate differences in lesions between the groups. Change in activity from baseline for each lesion identified was summarised by treatment group and overall and categorised as disappeared/decreased/showed no change/increased. Based on these appearances, a poor outcome was defined to have occurred in participants where lesions showed no change or had increased, and this was analysed by Fisher’s exact test. Following this analysis, a post hoc analysis was performed to evaluate the number of lesions that had disappeared between baseline and the end of study at patient level and lesion level using McNemar’s test. We attempted to establish whether there was a link between the location of lesions and the presence or severity of pain at that site at both baseline and the end of study using the BPI score. Analysis of changes in biochemical markers of bone turnover and serum alkaline phosphatase were modelled using a repeated measures analysis of covariance adjusting for the relevant baseline measure and the minimisation variables. The same approach was used to analyse changes in health-related quality of life (SF-36), pain (BPI) and HADS.

### Patient and public involvement

Patients were involved at the trial design stage when the methodology and endpoints were discussed. The Paget’s Association (a patient support group) was involved in publicising the study. The trial steering committee included a representative from the Paget’s Association and a person with Paget’s disease who had a family history of the disease. There was no patient or public involvement in data analysis or reporting of the trial.

## Results

**Figure 1:**
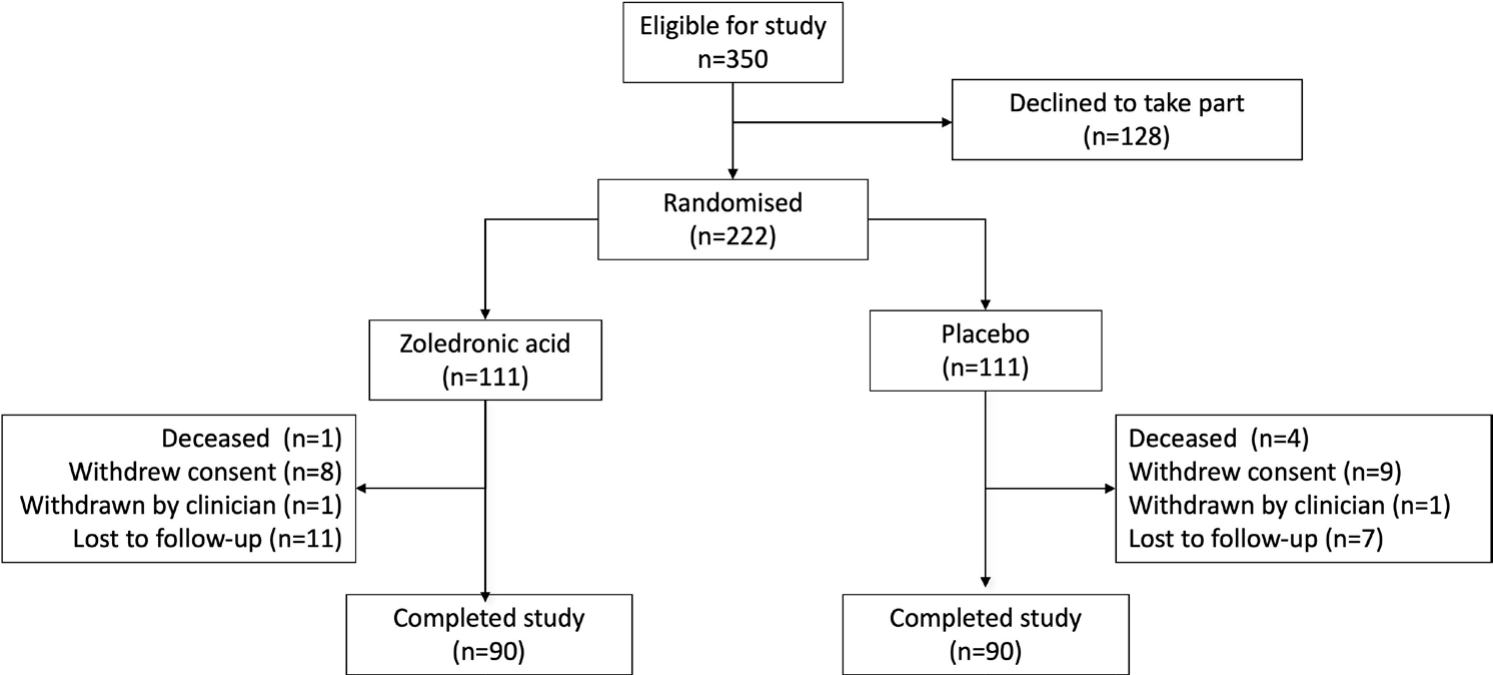
Disposition of study population.

Details of the programme of genetic testing of probands with PDB and their first degree relatives have previously been described in detail.^10 17^ In brief, the genetic testing programme identified 350 individuals who would have potentially been eligible to take part in the study, of whom 222 consented to enter the trial. Of these, 111 were randomised to receive a single intravenous infusion of ZA 5 mg and 111 to the infusion of placebo. Participants with serum 25(OH)D concentrations below 25 nmol/L at the baseline visit were treated with 100 000 units of cholecalciferol orally prior to receiving the infusion. Baseline characteristics of the study population are shown in table 1. The average age at entry to the study was about 50 years with a higher proportion of females than males. The proportion of current smokers was slightly higher in the placebo group. At baseline, the mean concentrations of biochemical markers of bone turnover lay within the reference range for all analytes tested. However, some individuals had elevated values. For total ALP, values were raised in four (3.6%) individuals in each group; for CTX, values were raised in two individuals in the ZA group (1.8%) and one individual (0.9%) in the placebo group. For PINP, values were raised in 19 (17.1%) individuals in the ZA group and 17 (15.3%) individuals in the placebo group. For BAP, 1 (0.9%) of participants in each group had raised values. Mean and median values for CTX, PINP, BAP and ALP at baseline were higher in those with bone lesions than those without lesions for each treatment group (online supplemental table 3). Serum 25(OH)D values were in the insufficient (25–50 nM) or normal range (>50 nM) in 201/222 (90.5%) of individuals. The most common variant in *SQSTM1* was p.Pro392Leu, occurring in about two thirds of individuals overall. Details of the individual variants by study group and their pathogenicity as assessed by the ACGS criteria^16^ are provided in online supplemental tables 4 and 5. The disposition of participants during the study is summarised in figure 1. The proportions of participants who were deceased, withdrew consent, were withdrawn by the clinician or who were lost to follow-up were similar in both groups and 90/111 (81%) participants in each group completed the study.

**Table 1:** Clinical and demographic details of study population

	Zoledronic acid (n=111)	Placebo (n=111)
Female	61 (55.0%)	60 (54.1%)
Age (years)	49.8 (8.8)	50.5 (9.3)
Current smoker	13 (11.7%)	20 (18.0%)
Regularly drinks alcohol	70 (63.1%)	71 (64.0%)
Body mass index	27.9 (5.3)	28.5 (6.3)
ALP (U/L)	78.2 (41.7)	80.1 (53.1)
Adjusted ALP*	0.44 (0.32)	0.47 (0.37)
Plasma CTX (µg/L)	0.33 (0.17)	0.35 (0.17)
Plasma PINP (µg/L)	55.0 (27.0)	59.5 (40.8)
Bone specific ALP (U/L)	11.0 (7.5)	9.9 (4.9–12.7)
Serum 25(OH) D (nmol/L)	66.7 (46.1)	64.9 (34.1)
Serum creatinine (µmol/L)	72 (13)	74 (13)
PDB lesions at baseline		
Monostotic	4 (3.6%)	5 (4.5%)
Polyostotic	5 (4.5%)	7 (6.3%)
Type of SQSTM1 mutation		
Missense	101 (91.0%)	101 (91.0%)
Truncating	10 (9.0%)	10 (9.0%)
Health-related quality of life		
BPI interference score	1.00 (1.71)	0.82 (1.49)
BPI severity score	1.34 (1.68)	1.24 (1.53)
SF-36 physical component	51.4 (8.1)	51.9 (8.6)
SF-36 mental component	52.5 (8.5)	52.5 (8.8)
HADS anxiety score	3.5 (2.7)	3.7 (3.2)
HADS depression score	3.3 (3.0)	3.5 (2.8)
HADS total score	6.9 (5.4)	7.3 (5.6)

Values are means (SD) or numbers and (%).

*Adjusted according to the local reference range in study centres (see methods). HADS anxiety and depression scores can range from 0 to 21 and total scores from 0 to 42. Higher scores indicate greater levels of anxiety and depression. SF-36 scores lower than 50 indicate lower quality of life and above 50 higher quality of life. The BPI score range from 1 to 10 with higher scores indicating more pain. For CTX, the unified reference range in men and women was 0.16–0.85 µg/L; for PINP the reference range was 15–76.3 µg/L and for BAP the reference range was 11.6–42.7 µg/L. Age-specific and sex-specific reference ranges are provided in online supplemental material.

ALP, alkaline phosphatase; Bone Specific ALP, bone-specific alkaline phosphatase; BPI, brief pain inventory; CTX, type I collagen C-terminal telopeptides; HADS, Hospital Anxiety and Depression Score; PDB, Paget’s disease of bone; PINP, procollagen type I amino-terminal propeptides; SF-36, short form 36.

### Bone lesions

Information on the effects of treatment on the evolution of bone lesions with the characteristics of PDB as assessed by bone scan is summarised in table 2. At baseline, 12 participants in the placebo group had PDB lesions compared with 9 in the ZA group. The number of individual lesions per participant ranged from 1 to 7. The total number of lesions was greater in the placebo group at baseline (29 vs 15). None of the participants allocated to receive ZA developed new lesions during the study compared with two in the placebo group, but this difference was not significant; (OR 0.41, 95% CI 0.00 to 3.43, p=0.247). There was a significant difference between the groups in the appearances of bone lesions between baseline and the end of study. Eight participants in the placebo group had a poor outcome (lesions which were new, unchanged or progressing) compared with none in the ZA group (OR 0.08, 95% CI 0.00 to 0.42, p=0.003). In the ZA group, nine participants had bone lesions at the start of the study compared with one participant at the end. This compares to 12 participants in the placebo group who had PDB lesions at the start of the study and 11 at the end (p=0.0034, between groups). Evolution of the appearances of lesions also differed significantly between the groups; in the ZA group, there were 15 lesions at baseline compared with two lesions at the end of study, compared with 29 lesions at baseline and 26 lesions at the end of study in the placebo group (p<0.0001 between groups). Participants who had bone lesions at baseline were on average, about 3 years older than those without lesions in both treatment groups (online supplemental table 3). There was no clear geographical pattern in participants who had lesions as compared with those that did not (online supplemental table 6)

**Table 2:** Lesions visualised by bone scan with the characteristics of Paget’s disease of bone

	Zoledronic acid (n=111)	Placebo (n=111)	OR (95% CI) p value
Interval between baseline and end of study radionuclide bone scans	78.4±24.5	79.0±24.3	
Participants with lesions at baseline	9 (8.1%)	12 (10.8%)	–
No of lesions per participant at baseline			
0	102 (91.9%)	99 (89.2%)	–
1	4 (3.6%)	5 (4.5%)	–
2	4 (3.6%)	3 (2.7%)	–
3	1 (0.9%)	1 (0.9%)	–
4	0 (0%)	2 (1.8%)	–
7	0 (0%)	1 (0.9%)	–
Total no of lesions at baseline	15	29	–
Participants with new lesions at the end of study	0 (0%)	2 (2.2%)	0.41 (0.0 to 3.43) 0.246
Participants with lesions at end of study*	1 (0.9%)	11 (9.9%)	0.0034
No of lesions at end of study	2	26	<0.0001
Poor outcome†	0	8	0.08 (0.0 to 0.42) 0.003

Bone scans were available for review at the end of study in 90 of the ZA group and 89 of the placebo group. The mean±SD interval between scans is provided in months.

*End of study values in the placebo group excluded one participant who had four lesions at baseline but who declined to have an end of study bone scan. This participant received rescue therapy with ZA during the study because of symptomatic Paget’s disease.

†Defined as emergence of new lesions, lesions remaining unchanged or progressed.

ZA, zoledronic acid.

### Skeletal events related to Paget’s disease

One participant who had been randomised to the placebo group had a skeletal event related to Paget’s disease. This participant had evidence of Paget’s disease affecting cervical vertebrae 3 and 4 on bone scan and X-ray at baseline (online supplemental figure 1). The participant entered the trial and was randomised to receive placebo. About 12 months into the study, the participant developed local pain and symptoms suggestive of nerve root compromise and was given ZA as rescue therapy with resolution of these symptoms.

### Biochemical markers of bone turnover

**Figure 2:**
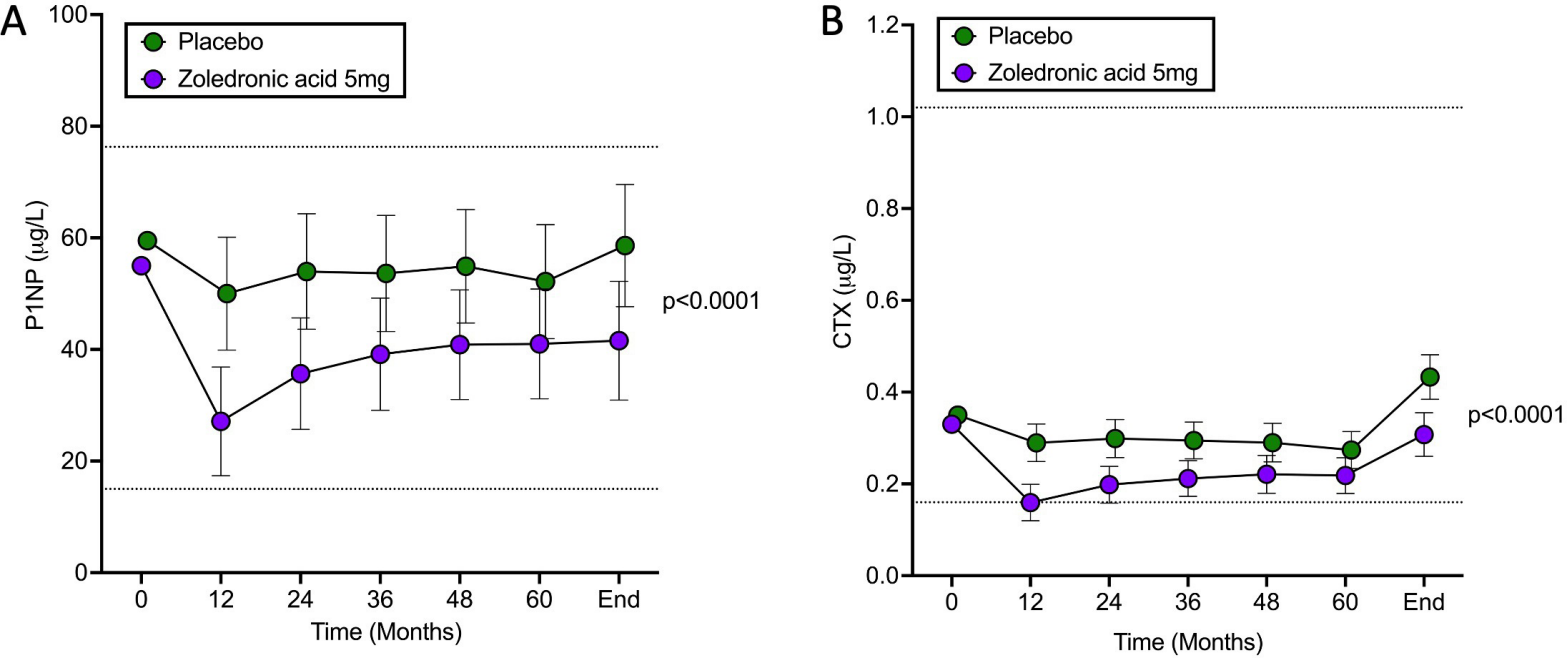
Changes in biochemical markers of bone turnover. The baseline values at month 0 are the means. Subsequent values are adjusted least squares means and 95% CIs. The p values refer to differences between the groups assessed by repeated measures ANOVA over the whole duration of the study. Unified reference ranges for men and women of all ages are indicated by the interrupted horizontal lines. Measurements of CTX were available in 103 of the ZA group at baseline, 100 at 12 months, 97 at 24 months, 96 at 36 months, 75 at 48 months, 62 at 60 months and 89 at the end of study. Corresponding values for the placebo group were 101, 97, 91, 93, 74, 50 and 89. For PINP, numbers in the ZA group were 103, 100, 97, 96, 75, 62 and 89; and in the placebo group, 101, 97, 91, 96, 74, 50 and 89. ANOVA, analysis of variance; CTX, type I collagen C-telopeptides; PINP, procollagen type I amino-terminal propeptides.

The effect of treatment on CTX, a biochemical marker of bone resorption and PINP, a biochemical marker of bone formation is shown in figure 2, panels A (PINP) and B (CTX). Plasma concentrations of CTX fell in the ZA group compared with baseline at 12 months and remained significantly lower in the ZA group compared with placebo throughout the study. The estimated least squares mean (95% CI) treatment difference taking all time points into account was −0.09 (−0.12 to –0.07), p<0.0001. The response of PINP was similar with an estimated treatment difference of −16.32 (−22.05 to –10.59), p<0.0001 in favour of the ZA group. Changes in BAP and total ALP are shown in online supplemental figure 2. Concentrations of BAP were significantly lower in the ZA group with an estimated treatment effect of −1.68 (−2.59 to –0.78), p=0.0003 and corresponding values for total ALP were −0.07 (−0.13 to –0.01), p=0.032. At the end of study visit, no individuals in the ZA group had raised CTX values compared with two individuals (2.2%) in the placebo group. Corresponding values for PINP were 3 (3.3%) vs 17 (18.9%); and corresponding values for BAP were 1 (1.1%) vs 1 (1.1%).

### Quality of life

The treatment groups were well matched at baseline in terms of quality of life, as assessed by the SF-36 physical component and mental component summary scores. The average values were close to 50 at baseline and remained stable throughout the study with no significant difference between treatment groups (online supplemental table 7). Similarly, the average BPI interference and severity scores were less than 2 at baseline in both groups and no significant differences were observed between treatment groups during the study (online supplemental table 7). The average HADS anxiety and depression scores were both less than 4 at baseline and no significant differences were observed between treatment groups throughout the study (online supplemental table 7).

### Adverse events

Data on adverse events are summarised in table 3. Overall, 77.3% of participants in the ZA group and 78.4% in the placebo group experienced at least one adverse event. Serious adverse events were recorded in 3.9% of the ZA group and 7.0% of the placebo group. Most of the adverse events were mild or moderate and only 5% in the ZA group and 2.8% in the placebo group were felt to be probably or definitely related to study medication by the local investigators. Adverse events related to the musculoskeletal system were those most commonly reported, followed by events related to infections and infestations (online supplemental table 8). The number of adverse events that were reported within the first 2 weeks following administration of study medication was similar between groups with 41 events in the ZA group and 39 in the placebo group.

**Table 3:** Adverse events

	Zoledronic acid (n=111)	Placebo (n=111)
All adverse events	583	644
Non-serious	560 (96.1%)	599 (93.0%)
Serious	23 (3.9%)	45 (7.0%)
Severity		
Mild	388 (66.6%)	389 (60.4%)
Moderate	180 (30.9%)	230 (35.7%)
Severe	15 (2.6%)	25 (3.9%)
Causality		
Unrelated	532 (91.3%)	597 (92.7%)
Possibly related	22 (3.8%)	29 (4.5%
Probably related	21 (3.6%)	16 (2.5%)
Definitely related	8 (1.4%)	2 (0.3%)

Values are numbers of events and per cent of events falling into a specific category.

## Discussion

The rationale for this study was to determine if it is possible to modify the natural history of PDB by early intervention with bisphosphonate therapy in people genetically at increased risk of developing the disease. We selected people who were carriers of pathogenic *SQSTM1* variants since these individuals have a high risk of developing PDB in later life^8^ and are more likely to develop severe disease and complications as compared with those who do not carry such variants.^9^ While bisphosphonates are known to be effective at suppressing raised bone turnover,^18^ restoring production of lamellar bone,^19^ improving the appearances of lytic lesions on X-ray^19^ and improving pain in people with PDB^18^ they have not yet been shown to halt progression, or reverse complications of the disease once these have developed.^4 5^

The primary endpoint of the study was to compare the proportion of individuals in each treatment group who developed new bone lesions. This was not met since only two participants developed new bone lesions during follow-up, but both occurred in the placebo group. The rate of emergence of new lesions was considerably less than expected. When choosing the sample size, we had anticipated that about 15% of individuals may have developed new lesions over a 5-year follow-up period but the actual rate was much less than that. It has previously been reported that approximately 80% of individuals with pathogenic variants in *SQSTM1* develop PDB by the seventh decade.^8^ In this study where the average age at entry was about 50 years, approximately 9% of participants had existing PDB lesions and those with bone lesions were approximately 3 years older than those without, emphasising the importance of increasing age as a risk factor for PDB. Further follow-up of this cohort in the ZIPP-Long Term Extension study (NCT03859895) is likely to provide new information on penetrance of the disease in these subjects as they get older.

One of the most striking findings to emerge from the study was the difference between groups on the evolution of existing bone lesions. In the ZA group, 13/15 existing lesions had completely disappeared on bone scan evaluation by the end of the study and the remaining two lesions had improved, whereas only one lesion disappeared in the placebo group. Reflecting this fact, only 1 participant in the ZA group had a visible lesion on bone scan by the end of the study compared with 11 in the placebo group. The number of bone lesions at the end of study was also significantly and substantially reduced in the ZA group compared with placebo. Minimisation was used to balance the groups for numbers of individuals with and without lesions at baseline. Although the proportion of participants with lesions was similar in the two groups, the number of lesions present in the placebo group at baseline was almost double that of the ZA group. We have no explanation for this other than to speculate that it occurred by chance since the baseline characteristics of each group were otherwise similar.

Changes in biochemical markers of bone turnover during the study also favoured ZA such that there was a significant reduction in the bone resorption marker CTX and the bone formation marker PINP over a median follow-up period of 84 months compared with placebo. There were also significant reductions in bone specific ALP and in total ALP in the ZA group, but the differences were less pronounced than for CTX and PINP. The intervention with ZA was well tolerated, and the total numbers of adverse events and serious adverse events were almost identical in the two groups. The prolonged suppressive effects on biochemical markers of bone turnover are in keeping with those previously reported by Reid *et al* who found that a single infusion of ZA suppressed bone turnover in people with established PDB for up to 78 months.^12^

Limitations of the study included the fact that 9% of individuals had evidence of PDB on bone scan at the outset of the study and that the proportion of individuals developing new bone lesions was much lower than expected. Both factors reduced the power to meet the primary outcome. While the study did not aim to investigate the effects of ZA on complications of PDB, one patient in the placebo group developed symptoms related to progression of PDB during the study and required rescue therapy with ZA. It is possible that the reversal of bone scan abnormalities by ZA may translate into clinical benefits in the longer term, but this can only be determined by longer-term follow-up of the study cohort.

The ZiPP study has for the first time demonstrated that it is both feasible and acceptable for people with a family history of PDB to undergo genetic testing for pathogenic *SQSTM1* variants and to offer these people a radionuclide bone scan to pick up early disease. We believe that a bone scan is an essential component of the proposed management pathway since our previous study demonstrated that biochemical markers of bone turnover had poor sensitivity and specificity in detecting PDB-like bone lesions in this patient group.^17^ The study has also shown that a single infusion of ZA is well tolerated and that it can favourably modify the raised bone turnover that is characteristic of active disease, as reflected by bone scan appearances and biochemical markers of bone turnover.

Although there was no significant difference between groups in the emergence of new lesions and skeletal adverse events related to PDB, the effect size of ZA noted in this study is similar to that observed with adjuvant bisphosphonate therapy in the Early Breast Cancer Trialists’ Collaborative Group meta-analysis.^20^ This has led to the widespread introduction of adjuvant ZA therapy for postmenopausal women with early breast cancer as part of standard care. We believe that a similar approach is now justified in people with a family history of PDB who test positive for *SQSTM1* mutations. At the present time, it remains to be determined whether it would be appropriate to offer all carriers of pathogenic *SQSTM1* variants treatment with ZA or whether therapy would be better targeted only to those with lesions. Future research to gather the views of people with a family history of PDB will help to inform the most appropriate way forward.

## Data Availability

Data are available on reasonable request.

## Data availability statement

The datasets generated and analysed during this clinical trial are not yet publicly available. It is anticipated that an anonymised dataset will be made available to bona fide researchers subject to completion of an appropriate data sharing agreement provided the proposed use of the data is in accordance with the informed consent provided by study participants.
