# Supplementary material for: Randomised trial of genetic testing and targeted intervention to prevent the development and progression of Paget’s disease of bone
Source: Ann Rheum Dis. 2023 Dec 20;83(4):529–36. doi: 10.1136/ard-2023-224990 (PMC10958267; doi:10.1136/ard-2023-224990)
Supplement: Supplementary data [file ard-2023-224990supp001.pdf]

Supplementary Material

Randomised trial of genetic testing and targeted intervention to prevent the development and progression of Paget's disease of Bone.

Jonathan Philips PhD[1], Deepak Subedi MB ChB [2], Steff C Lewis PhD[3], Catriona Keerie PhD[3], Owen Cronin PhD[1 †], Mary Porteous MD [4], David Moore [4], Roseanne Cetnarskyj PhD [5], Lakshminarayan R Ranganath MD, PhD [6], Peter L Selby MD, [7], Tolga Turgut MD[7], Geeta Hampson MD [8], Rama Chandra MB BS[9], Shu Ho MD [10], Jon H Tobias MD, PhD[11], Steven Young Min PhD [12], Malachi McKenna MD[,13], Rachel Crowley MD [13], William D. Fraser MD[14], Jonathan C.Y. Tang PhD [14,15], Luigi Gennari MD PhD[16], Rannuccio Nuti MD [16], Maria-Luisa Brandi MD PhD[17], Javier del Pino-Montes MD [18], Jean-Pierre Devogelaer MD [19], Anne Durnez MD PhD[19], Giovanni Carlo Isaia MD[20], Marco Di Stefano MD[20], Nuria Guanabens MD[21,] Josep Blanch Rubio MD[22], Markus J Seibel MD PhD[23], John P Walsh PhD[24,25], Sarah Rea PhD [24,25 ‡], Mark A Kotowicz MB BS [26] Geoffrey C Nicholson PhD [27], Emma L Duncan PhD [28 §], Gabor Major MB BS [29,30], Anne Horne MB, ChB [31], Nigel Gilchrist MB ChB[32], Stuart H. Ralston MD[1]

Technical Methods

Routine Biochemistry

Blood samples for routine biochemistry were analysed at the local hospital laboratories of participating centres and measurements were made of serum creatinine, serum total alkaline phosphatase (ALP), calcium, albumin, liver function tests and 25(OH)D according to standard techniques. Measured ALP values were adjusted to the local reference range (RR) to give an adjusted ALP value which was used in statistical analysis. The formula used for calculation was as follows: Adjusted ALP = (measured ALP-ALP lower limit of RR) / (ALP upper limit of RR – ALP lower limit of RR). According to this methodology, a value of 1.0 would equate to an ALP at the upper limit of the reference range, a value of 2.0 to twice the upper limit; a value of 0.5 to the mid-point of the reference range and a value of 0 the lower limit of the reference range.

Specialised markers of bone resorption and formation

Measurement of these markers were performed at the University of East Anglia. Blood samples for the markers were obtained between 09.00 and 12.00. Measurements of Type I collagen C-telopeptides (CTX) and Procollagen type I amino-terminal propeptide (PINP) were made on plasma separated from whole blood collected into tubes containing potassium EDTA as an anticoagulant. Bone specific alkaline phosphatase (BAP) was measured on serum separated from whole blood.

Measurements of plasma CTX were made using an Electrochemiluminescence immunoassay (ECLIA) on a Cobas e601 analyser (Roche Diagnostics, Germany). The inter-assay coefficient of variation (CV) for CTX was ≤3% between 0.2 and 1.5 μg/L with the sensitivity of 0.01 μg/L. The reference ranges in women were; 0.16-0.57 μg/L (age<50); 0.25-1.02 μg/L (age>50) and in men were; 0.19-0.58 μg/L (age 30-50), 0.19-0.70 μg/L (age 50-70), and 0.19-0.85 μg/L (age >70). Measurements of PINP were also made by ECLIA on a Cobas e601 analyser. The P1NP inter-assay CV was ≤3% between 20-600 μg/L with the sensitivity of 8 μg/L. The reference range in premenopausal women was 15-58.6 μg/L; in post-menopausal women 20.3-76.3 μg/L, and in men 20-76 μg/L). Serum bone-specific alkaline phosphatase (BAP) was measured using the MicroVue enzyme immunoassay (Quidel, Athens, OH, USA). Inter-assay CV for BAP was ≤2.4% up to the concentration of 140 U/L with the lower limit of sensitivity at 0.7 U/L. The reference ranges in pre-menopausal women were 11.6-29.6 U/L, in post-menopausal women 14.2-42.7 U/L and in men 15-41.2 U/L).

Supplementary Table 1. Country and site-specific ethical approvals

Country	Site	REC reference	REC Name
Australia	Brisbane	HREC/12/QRBW/199	Royal Brisbane & Women's Hospital Human Research Ethics Committee (HREC)
Australia	Geelong	46631	Barwon Health Human Research Ethics Committee (HREC)
Australia	Newcastle Sydney	2019/ETH07835	Concord Repatriation General Hospital (CRGH) Human Research Ethics Committee (HREC)
Australia	Perth	RGS0000001553	Sir Charles Gairdner Group (SCGG) Human Research Ethics Committee (HREC)
Australia	Toowoomba	#12/04	St Vincent’s Health and Aged Care Human Research Ethics Committee (HREC)
Belgium:	Brussel	2010/050CT/308	Comité d'Éthique Hospitalo-Facultaire
Ireland:	Dublin	ZiPP Trial	SVHG Ethics and Medical Research Committee
Italy	Siena	2008-005667-34	Comitato Etico Locale per la Sperimentazione Clinica Dei Medicinal dell'Azienda Ospideliera Universitaria seenese di Siena
Italy	Turin	2008-005667-34	Comitato etico dell Azienda Ospedaliera Universitaria S Giovanni Battista Di Torino
Italy	Florence	2008-005667-34	Comitato etico dell Azienda Ospedaliera Universitaria Careggi
New Zealand:	Auckland	13/STH/32/AM13	Southern Health and Disability Ethics Committee
New Zealand	Christchurch	13/STH/32/AM13	Southern Health and Disability Ethics Committee
Spain:	Salamanca	09/813	CEIM Área de Salud de Salamanca
Spain:	Barcelona	09/813	CEIM Área de Salud de Salamanca

Supplementary Table 2. Participating centres

**Liverpool**<br>**Principal Investigator**<br><br>Department of Clinical Biochemistry and Metabolic Medicine,<br>The University of Liverpool<br>Liverpool<br>L69 3GA<br>United Kingdom<br>Tel: 0151 706 4197<br>Email:	**Manchester**<br>**Principal Investigator**<br>Email: peter.selby@mft.nhs.uk
**London Guys**<br>**Principal Investigator**<br>Dr Geeta Hampson<br>Department of Rheumatology,<br>St. Thomas' Hospital,<br>London,<br>SE1 7EH<br>United Kingdom<br>Tel: 0207 188 1284<br>Email: geeta.hampson@kcl.ac.uk	**Oswestry**<br>**Principal Investigator**<br>Dr Shu Ho<br>The Robert Jones and Agnes Hunt Orthopaedic and District Hospital,<br>Oswestry,<br>SY10 7AG<br>United Kingdom<br>Tel: 01691 404354<br>Email: Shu.Ho@nhs.net
**London Kings College**<br>**Principal Investigator**<br>Dr Rama Chandra<br>Clinical Biochemistry,<br>King's College Hospital,<br>London,<br>SE5 9RS<br>United Kingdom<br>Tel: 0203 299 3275<br>Email: rama.chandra@nhs.net	**Portsmouth**<br>**Principal Investigator**<br>Dr Steven Young Min<br>Department of Rheumatology,<br>Queen Alexandra Hospital,<br>Portsmouth,<br>PO6 3LY<br>United Kingdom<br>Tel: 02392 286199<br>Email: Steven.YoungMin@porthosp.nhs.uk
**Bristol**<br>**Principal Investigator**<br>Professor Jon Tobias<br>Musculoskeletal Research Unit,<br>University of Bristol,<br>BS10 5NB<br>United Kingdom<br>Tel: 02392 286199<br>Email: jon.tobias@bristol.ac.uk	**Norwich**<br>**Principal Investigator**<br>Professor William Fraser<br>Norwich Medical School,<br>Faculty of Medicine and Health Sciences,<br>University of East Anglia,<br>Norwich,<br>NR4 7TJ,<br>United Kingdom<br>Tel: 01159 691 169<br>Email: w.fraser@uea.ac.uk

**Wrexham**<br>**Principal Investigator**<br>Dr Mark Garton<br>Betsi Cadwaladr University Health Board /Bwrdd Iechyd Prifysgol Betsi<br>Wrexham Technology Park<br>Wrexham<br>LL13 7YP<br>United Kingdom<br>Tel: 03000 858034<br>Email: Mark.Garton@wales.nhs.uk	**Dublin**<br>**Principal Investigators**<br>Dr Malachi Mckenna / Dr Rachel Crowley<br>Endocrinology Department,<br>St. Vincent's University Hospital,<br>Dublin, 4<br>Republic of Ireland<br>Tel: 01-2214407<br><br>Email: malachimckenna@gmail.com; rcrowley@svhg.ie
**Norwich**<br>**Principal Investigator**<br>Dr Jonathan Tang<br>Norwich Medical School,<br>Faculty of Medicine and Health Sciences,<br>University of East Anglia,<br>Norwich, NR4 7TJ,<br>United Kingdom<br>Tel: 01159 691 169<br>Email: Jonathan.Tang@uea.ac.uk	**Siena**<br>**Principal Investigators**<br>Dr Luigi Gennari / Professor Rannuccio Nuti<br>Department of Medicine,<br>Surgery and Neurosciences,<br>University of Sienna,<br>Sienna,53100<br>Italy<br>Tel: 0577 585 362<br>Email: gennari@unisi.it; nutir@unisi.it
**Hospital Clinic, Barcelona**<br>**Principal Investigator**<br>Dr Nuria Guanabens<br>Department of Rheumatology<br>Hospital Clinic,<br>CIBERehd<br>Barcelona 8036<br>Spain<br>Tel: 34932275400<br>Email: nguanabens@ub.edu	**Hospital Del Mar, Barcelona**<br>**Principal Investigator**<br>Dr Josep Blanch<br>Bone Metabolism Unit<br>Rheumatology Division,<br>University Hospital del Mar<br>Barcelona 8003<br>Spain<br>Tel: 34933674126<br>Email: jblanch@parcdesalutmar.cat
**Salamanca**<br>**Principal Investigators**<br>Dr Ana Isabel Turrión Nieves<br>Dr Javier del Pino-Montes<br>Institute for Biomedical Research<br>University Hospital of Salamanca<br>Salamanca 37007<br>Spain<br>Tel: 697 50 43 76<br>Email: anaturrion@usal.es; jpino@usal.es	**Florence**<br>**Principal Investigator**<br>Professor Maria Brandi<br>Department of Internal Medicine, University Hospital of Careggi,<br>Florence,<br>50139<br>Italy<br>Tel: 0554 296 586<br>Email: m.brandi@dmi.unifi.it

**Turin**<br>**Principal Investigators**<br>Professor Giancarlo Isaia / Professor Marco Di Stefano<br>Geriatrics and Metabolic Bone Diseases, AOU San Giovanni Battista di Torino Corso, Torino, 10126<br>Italy<br>Tel: 390116637140<br>Email: giancarlo.isaia@unito.it; mdistefano@cittadellasalute.to.it	**Brussels**<br>**Principal Investigators**<br>Dr Anne Durnez / Dr Jean-Pierre Devogelaer<br>Service de Rhumatologie<br>Cliniques Universitaires Saint-Luc,<br>Brussels, B-1200<br>Belgium<br>Tel +32 2 764 5390<br>Email: anne.durnez@uclouvain.be; jean-pierre.devogelaer@uclouvain.be
**Sydney Concord**<br>Principal Investigator<br>Professor Markus Seibel<br>Department of Endocrinology and Metabolism,<br>Concord Repatriation General Hospital,<br>Sydney,<br>NSW 2139<br>Australia<br>Tel: 0061 297 676 109<br>Email: Markus.seibel@sydney.edu.au	**Geelong**<br>Principal Investigator<br>Prof Mark Kotowicz<br>Department of Endocrinology and Diabetes,<br>The University of Melbourne,<br>Geelong,<br>3220<br>Australia<br>Tel: 0061 352 267 764<br>Email: MARKK@barwonhealth.org.au
**Perth**<br>Principal Investigator<br>Dr John Walsh<br>Department of Endocrinology and Diabetes,<br>Sir Charles Gairdner Hospital,<br>Perth,<br>6009<br>Australia<br>Tel: 0061 893 462 466<br>Email: John.Walsh@health.wa.gov.au	**Brisbane**<br>Principal Investigator<br>Professor Emma Duncan<br>Endocrinology Department,<br>Royal Brisbane and Woman's Hospital, Brisbane,<br>4029<br>Australia<br>Tel: 0061 7 3636 8111<br>Email: emma.duncan@qut.edu.au
**Toowoomba**<br>Principal Investigator<br>Professor Geoffrey Charles Nicholson<br>Rural Clinical School Research Centre, The University of Queensland, Queensland,<br>4350<br>Australia<br>Tel: 00617 4633 9702<br>Email: geoffn@geelongendo.com.au	**Auckland**<br>Principal Investigator<br>Dr Anne Horne<br>Bone & Joint Research Group<br>Department of Medicine,<br>University of Auckland,<br>Auckland,<br>New Zealand<br>Tel: (09) 923 9787<br>Email: a.horne@auckland.ac.nz

**Newcastle**<br>Principal Investigator<br>Dr Gabor Major<br>Rheumatology,<br>Bone and Joint Institute,<br>Royal Newcastle Centre,<br>New Lambton Heights,<br>2305<br>Australia<br>Tel: 02 4922 3500<br>Email:<br>Gabor.Major@hnehealth.nsw.gov.au	**Christchurch**<br>Principal Investigator<br>Dr Nigel Gilchrist<br>The CGM Research Trust,<br>Christchurch,<br>8022<br>New Zealand.<br>Tel: 64 333 77820<br>Email: nigel.gilchrist@cgm.org.nz

Supplementary Table 3. Baseline Characteristics of groups with and without bone lesions at baseline

	Zoledronic Acid (N=111)		Placebo (N=111)	
	Lesion (n=9)	No Lesion (n=102)	Lesion (n=12)	No Lesion (n=99)
Female Gender	4 (44.4%)	57 (55.9%)	6 (50.0%)	54 (54.5%)
**Type of mutation**				
Missense	8 (88.9%)	93 (91.2%)	12 (100.0%)	89 (89.9%)
Truncating	1 (11.1%)	9 (8.8%)	0 (0.0%)	10 (10.1%)
**Age band**				
30 - 40 years	0 (0.0%)	15 (14.7%)	0 (0.0%)	14 (14.1%)
41 - 50 years	4 (44.4%)	41 (40.2%)	5 (41.7%)	42 (42.4%)
51 - 60 years	3 (33.3%)	36 (35.3%)	5 (41.7%)	31 (31.3%)
61 - 70 years	2 (22.2%)	8 (7.8%)	1 (8.3%)	8 (8.1%)
71+ years	0 (0.0%)	2 (2.0%)	1 (8.3%)	4 (4.0%)
**Age (years)**				
Mean (SD)	52.7 (9.3)	49.6 (8.8)	53.2 (8.8)	50.2 (9.4)
Median [Q1-Q3]	52.0 [46-55]	49.5 [43-56]	51.0 [49-55]	50.0 [43-56]
Min - Max	41 , 68	32 , 74	41 , 75	32 , 75
**CTX (ng/mL)**				
N (missing)	8 (1)	95 (7)	10 (2)	91 (8)
Mean (SD)	0.40 (0.19)	0.32 (0.17)	0.42 (0.32)	0.34 (0.14)
Median [Q1-Q3]	0.42 [0.27-0.55	0.30 [0.20-0.37	0.37 [0.23-0.44	0.32 [0.24-0.44
Min - Max	0.08 , 0.61	0.09 , 1.24	0.14 , 1.28	0.06 , 0.70
**P1NP (ng/mL)**				
N (missing)	8 (1)	95 (7)	10 (2)	91 (8)
Mean (SD)	77.8 (34.0)	53.1 (25.7)	104.8 (110.8)	54.6 (19.2)
Median [Q1-Q3]	93.5 [47-106]	48.2 [38-65]	71.8 [56-102]	51.3 [43-63]
Min - Max	22 , 107	21 , 217	38 , 412	21 , 127
**BALP (U/L)**				
N (missing)	8 (1)	95 (7)	10 (2)	90 (9)
Mean (SD)	14.1 (7.6)	10.8 (7.4)	14.0 (16.5)	10.2 (6.5)
Median [Q1-Q3]	15.9 [10-20]	9.1 [6-13]	9.6 [4-12]	9.9 [5-13]
Min - Max	0 , 22	1 , 52	3 , 58	1 , 36
**ALP (U/L)**				
N (missing)	9 (0)	102 (0)	12 (0)	99 (0)
Mean (SD)	95.0 (34.8)	76.8 (42.1)	131.7 (144.6)	73.9 (21.2)
Median [Q1-Q3]	88.0 [66-113]	72.0 [60-83]	81.0 [65-108]	70.0 [58-86]
Min - Max	58 , 151	16 , 446	60 , 569	24 , 137

Supplementary Table 4. Individual pathogenic variants by study group.

	Zoledronic Acid (N=111)	Placebo (N=111)
**Type of Mutation**		
Missense	101 (91.0%)	101 (91.0%)
Truncating	10 (9.0%)	10 (9.0%)
**Amino Acid Change**		
c.1165+1G>A*	5 (4.5%)	3 (2.7%)
p.Thr350GlnfsTer28	2 (1.8%)	1 (0.9%)
p.Glu396Ter	1 (0.9%)	3 (2.7%)
p.Gln371Ter	1 (0.9%)	1 (0.9%)
p.Lys378Ter	1 (0.9%)	1 (0.9%)
p.Pro392Leu	64 (57.7%)	77 (69.4%)
p.Met404Val	13 (11.7%)	12 (10.8%)
p.Phe406Val	2 (1.8%)	0 (0.0%)
p.Gly411Ser	7 (6.3%)	2 (1.8%)
p.Ile424Ser	2 (1.8%)	0 (0.0%)
p.Gly425Arg	13 (11.7%)	11 (9.9%)

Values are numbers and percentages. *This is a mutation at a canonical splice donor site at the start of intron 7 which is predicted to disrupt splicing resulting in a truncated protein of 390 amino acids.

Supplementary Table 5. Pathogenicity of variants assessed by ACGS best practice guidance

	Allele Frequency			
**Amino Acid Change**	**Study**	**GnomAD**	**ACGS Criteria**	**Pathogenicity**
c.1165+1G>A*	0.011	1.36E-05	PVS1_str, PS4, PM1, PP1_str	Pathogenic
p.Thr350fsGlnfsTer28	0.004	4.10E-06	PVS1_str, PS4, PM1, PP1_str	Pathogenic
p.Glu396Ter	0.001	4.10E-06	PVS1_str PS4, PM1, PP1_str	Pathogenic
p.Gln371Ter	0.003	3.60E-06	PVS1_str, PS4, PM1, PP1_str	Pathogenic
p.Lys378Ter	0.003	8.40E-06	PVS1_str, PS4, PM1, PP1_str	Pathogenic
p.Pro392Leu	0.188	0.0014	PP2, PS3_mod, PS4, PM1, PP1_str	Pathogenic
p.Met404Val	0.033	2.91E-05	PP2, PS3_mod, PS4, PM1, PP1_str	Pathogenic
p.Phe406Val	0.003	3.42E-06	PP2, PS3_mod, PS4, PM1, PP1_str	Pathogenic
p.Gly411Ser	0.012	6.13E-05	PP2, PS3_mod, PS4, PM1, PP1_str	Pathogenic
p.Ile424Ser	0.003	9.60E-06	PP2, PS3_mod, PS4, PM1, PP1_str	Pathogenic
p.Gly425Arg	0.032	3.15E-05	PP2, PS3_mod, PS4, PM1, PP1_str	Pathogenic

The frequency of individual variants in the study population are shown in relation to the frequency in the GnomAD database. The degree of enrichment in the study population over GnomAD ranged from 132-fold for pPro392Leu to 1114-fold for p.Met404Val. Pathogenicity was assessed by the UK Association for Clinical Genomic Science (ACGS) best practice guidelines for variant classification in rare disease [1], which in turn were based on the consensus recommendations of the American College of Medical Genetics and Genomics and the Association for Molecular Pathology [2]
.

Supplementary Table 6. Baseline lesions by country of randomisation

Country	Baseline lesion	No baseline lesion
Australia	3 (8.8%)	31 (91.2%)
UK	11 (8.2%)	123 (91.8%)
Ireland	0 (0.0%)	10 (100.0%)
New Zealand	1 (25.0%)	3 (75.0%)
Spain	4 (22.2%)	14 (77.8%)
Belgium	0 (0.0%)	3 (100.0%)
Italy	2 (10.5%)	17 (89.5%)

Values are numbers (%).

Supplementary Figure 1. Images of participant who developed a Paget's disease related skeletal event.

A

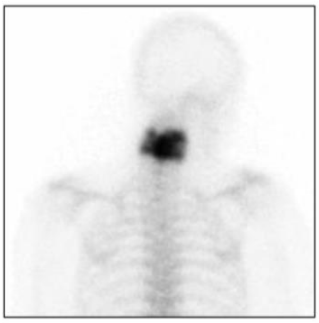

B

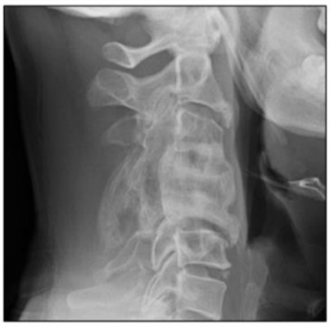

Panel A. Radionuclide bone scan image showing increased tracer uptake in the cervical spine. Panel B. Radiograph showing expansion of C4 and C5 typical of Paget's disease. The participant was symptom free at baseline but developed symptoms related to Paget's of the cervical spine after about 1 year and required rescue therapy with zoledronic acid.

Supplementary Table 7. Quality of Life Questionnaire scores

	Zoledronic Acid (N=111)	Placebo (N=111)	Mean [95% CI] p-value
**Health Anxiety and Depression Scale (HADS) - Anxiety Score**			
Baseline	3.5	3.7	-0.19 [-0.87- 0.49] p-value 0.574
End of Study	3.3	3.7	
**Health Anxiety and Depression Scale (HADS) - Depression Score**			
Baseline	3.3	3.5	-0.29 [-0.90 - 0.31] p-value 0.340
End of Study	3.1	3.6	
**Brief Pain Inventory - Interference Score**			
Baseline	1.00	0.82	-0.37 [-0.78 - 0.03] p-value 0.070
End of Study	0.98	1.21	
**Brief Pain Inventory - Severity Score**			
Baseline	1.34	1.24	-0.28 [-0.70- 0.13] p-value 0.175
End of Study	1.38	1.75	
**SF-36 - Physical Component Score**			
Baseline	51.4	51.9	1.60 [-0.24 - 3.43] p-value 0.086
End of Study	51.7	49.7	
**SF-36 - Mental Component Score**			
Baseline	52.5	52.5	0.51 [-1.31- 2.32] p-value 0.584
End of Study	53.3	51.8	

Baseline values are means, the end of study values are adjusted least squares means with 95% confidence intervals. HADS anxiety and depression scores can range from 0-21. Higher scores indicate greater levels of anxiety and depression. The BPI score range from 1-10 with higher scores indicating more pain. For SF36 scores lower than 50 indicate lower quality of life and above 50, a higher quality of life.

Supplementary Table 8. Adverse events grouped by study group.

	Zoledronic Acid (n=111)	Placebo (n=111)	Total
Total Adverse Events	583	644	1,227
Blood and lymphatic system disorders	0 (0.0%)	3 (0.5%)	3 (0.2%)
Cardiac disorders	3 (0.5%)	4 (0.6%)	7 (0.6%)
Congenital, familial and genetic disorders	0 (0.0%)	1 (0.2%)	1 (0.1%)
Ear and labyrinth disorders	6 (1.0%)	9 (1.4%)	15 (1.2%)
Endocrine disorders	4 (0.7%)	3 (0.5%)	7 (0.6%)
Eye disorders	5 (0.9%)	6 (0.9%)	7 (0.6%)
Gastrointestinal disorders	30 (5.1%)	47 (7.3%)	77 (6.3%)
General disorders and administration site conditions	10 (1.7%)	21 (3.3%)	31 (2.5%)
Hepatobiliary disorders	0 (0.0%)	6 (0.9%)	6 (0.5%)
Immune system disorders	2 (0.3%)	1 (0.2%)	3 (0.2%)
Infections and infestations	149 (25.6%)	116 (18.0%)	265 (21.6%)
Injury, poisoning and procedural complications	38 (6.5%)	51 (7.9%)	89 (7.3%)
Investigations	45 (7.7%)	57 (8.9%)	102 (8.3%)
Metabolism and nutrition disorders	8 (1.4%)	11 (1.7%)	19 (1.5%)
Musculoskeletal and connective tissue disorders	97 (16.6%)	110 (17.1%)	207 (16.9%)
Neoplasms benign, malignant and unspecified (including cysts and polyps)	12 (2.1%)	7 (1.1%)	19 (1.5%)
Nervous system disorders	36 (6.2%)	31 (4.8%)	67 (5.5%)
Pregnancy, puerperium and perinatal conditions	0 (0.0%)	2 (0.3%)	2 (0.2%)
Product issues	0 (0.0%)	0 (0.0%)	0 (0.0%)
Psychiatric disorders	10 (1.7%)	17 (2.6%)	27 (2.2%)
Renal and urinary disorders	4 (0.7%)	10 (1.6%)	14 (1.1%)
Reproductive system and breast disorders	14 (2.4%)	16 (2.5%)	30 (2.4%)
Respiratory, thoracic and mediastinal disorders	10 (1.7%)	18 (2.8%)	28 (2.3%)
Skin and subcutaneous tissue disorders	9 (1.5%)	17 (2.6%)	26 (2.1%)
Social circumstances	0 (0.0%)	2 (0.3%)	2 (0.2%)
Surgical and medical procedures	86 (14.8%)	68 (10.6%)	154 (12.6%)
Vascular disorders	5 (0.9%)	10 (1.6%)	15 (1.2%)

The adverse events are categorised by the Medical Dictionary for Regulatory Activities – (MedDRA) system organ classes. Values are number and percentages for the events reported.

Supplementary Figure 2. Changes in alkaline phosphatase and bone specific alkaline phosphatase

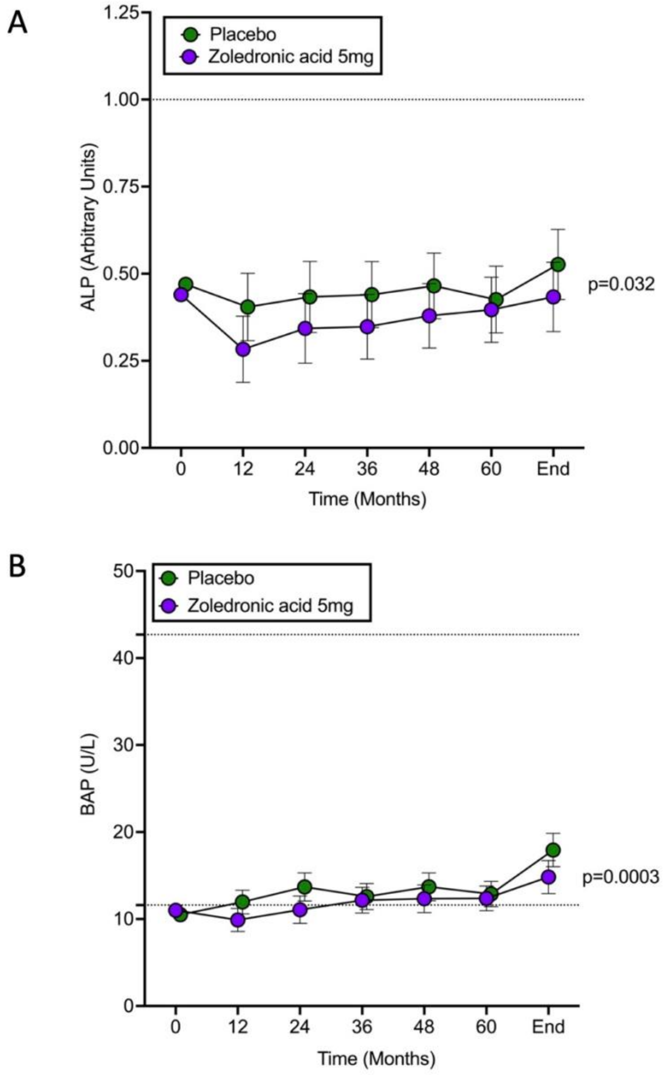

Panel A shows adjusted alkaline phosphatase (ALP). Panel B shows Bone-Specific Alkaline Phosphatase (BAP). Baseline values are means. Subsequent values are adjusted least squares means and 95% confidence intervals. The p-values refer to differences between groups by repeated measures ANOVA taking all observations into account. The unified reference ranges are indicated by horizontal interrupted lines. The baseline values are the means. Subsequent values are adjusted least squares means and 95% confidence intervals. The p-values refer to differences between the groups assessed by repeated measures ANOVA over the whole duration of the study. Unified reference ranges for men and women of all ages are indicated by the interrupted horizontal lines. Measurements of ALP were available at baseline in 103 of the ZA group, 100 at 12 months, 97 at 24 months, 96 at 36 months, 75 at 48 months, 62 at 60 months, and 89 at the end of study. Corresponding values for the placebo group were 101; 97, 91, 93, 74, 50 and 89. Corresponding numbers for BAP in the ZA group were 103, 98, 97, 97, 74, 62, 88 and for placebo group were 100; 97, 91, 93, 75, 51 and 89.

References

1. Ellard S BE, Callaway A, Berry A, Forrester N, Turnbulll C, Owens M, Eccles DM, Abbs R, Scott R, Deans ZC, Lester T, Campbell J, Newman WG, Ramsden S, McMullan DJ. ACGS best practice guidelines for Variant Classification in Rare Disease 2020. 2020 [cited 2023; Available from: https://www.acgs.uk.com/media/11631/uk-practice-guidelines-for-variant-classification-v4-01-2020.pdf

2. Richards S, Aziz N, Bale S, *et al.* Standards and guidelines for the interpretation of sequence variants: a joint consensus recommendation of the American College of Medical Genetics and Genomics and the Association for Molecular Pathology. Genet Med. 2015; 17:405-424. doi:10.1038/gim.2015.30
